# Little patients, large risks: An overview on patient safety management in pediatrics settings

**DOI:** 10.3389/fped.2022.919710

**Published:** 2022-09-16

**Authors:** Simona Nicolì, Marcello Benevento, Davide Ferorelli, Gabriele Mandarelli, Biagio Solarino

**Affiliations:** ^1^Section of Legal Medicine, Interdisciplinary Department of Medicine, University of Bari, Bari, Italy; ^2^Section of Forensic Psychiatry, Interdisciplinary Department of Medicine, University of Bari, Bari, Italy

**Keywords:** children safety, pediatric clinical pathways, clinical risk management, children's hospital, errors

## Introduction

Patient safety is an emerging healthcare discipline with the ultimate goal to reduce errors and harm to patients by implementing quality health services. In 1999, the well-known “To Err is Human: Building a safer health system” reported that between 2% and 4% of people die annually in United States hospitals for medical errors (1). Since that publication, the focus on healthcare safety has encouraged efforts by legislators, hospital government, and health professionals to promote policies and behaviors heavily to reduce errors and implement a safe provision of healthcare delivery. In 2019, the World Health Organization (WHO) declared September 17th as Patient Safety Day, confirming that as a global priority (2).

The increasing interest in patient safety has significant repercussions on scientific publications with an ever-increasing production of studies in the growing interdisciplinary field of public health. New concepts and new terms had born, such as “clinical risk,” which refers to the probability that a patient can be the victim of an adverse event due to medical care, although unintentionally.

The WHO proposed to adopt a “universal vocabulary” thus standardizing the terminology and allowing effective scientific research. In clinical risk, the most explored field is adult patient safety, where growing interest is shown primarily to prevent nosocomial infections, falls, and pressure injuries. Instead, a little-explored area concerns the pediatric population (3).

This article aims to overview the spread of pediatric clinical risk management and organizational culture for healthcare quality improvement, looking at what has been done and enhancing healthcare practices to implement inpatient safety. The authors have reviewed the main concerns on pediatric patient safety and issued the main medico-legal aspects. After summarizing the relevant literature, the authors addressed their point of view by writing an opinion article.

## Pediatric patient safety: The main issues

Nowadays, we are witnessing a growing interest in errors and harm in the pediatric setting, which differ from adults. Standard textbooks and research literature still give little attention to pediatric inpatient primary care and well-being. The short amount available of scientific works is due to children's particular features, characterizing their health management as different and more complex than adults. A study published in 2005 reported that adverse events occurred in children in 1% of pediatric hospitalizations and that 60% of these events were preventable. Other studies suggest that this rate may be higher (4). Table 1 summarizes the main facets of pediatric patient safety.

**Table 1:** Major concerns regarding pediatric patient safety.

**Matter of concern**	**Issues**
Physical characteristics	Children's small size, weight, and morphology need a suitable healing environment (5, 6)
Capacity to consent to medical treatment/Legal Status	Cognitive development affects the capacity to choose a medical treatment/ consent to clinical research. The assessment of decision-making capacity in minors is one of the most compelling and debated topics in pediatric healthcare (7–10). The legal status of minors slightly changes between Countries. Normally, caregivers' permission is needed to choose or refuse any kind of treatment (11)
Lack of children-dedicated healthcare facilities	Settings in which patients had the most complex needs imply a high error rate. Children require healthcare focusing on their needs so they must be treated in children's hospitals (12)
Lack of pediatric guidelines	The growing lawsuits against healthcare professionals led the scientific community to great interest in drafting clinical guidelines, which have to be standardized and subjected to quality control in recent years (13, 14)
Lack of labeled medications	Most medications are not labeled for use in the pediatric population (15)

### The assessment of decision-making capacity

Woods highlighted children's physical characteristics can affect the predisposition to errors and harm: weight-based medication dosing, significant variation in size and weight, and predisposition to medical error (5). Moreover, the number of children with chronic diseases and the obesity rate increases, so the physical features of children are sort of changing (16).

It is not unusual for physicians to ask for legal intervention when they believe that a caregiver's refusal of treatment places patients at substantial risk. This phenomenon became most dramatic in pediatric oncology, where the potential need to override caregivers' decision-making when they refuse treatment or replace it with ineffectual alternatives is well-known. Hence the question: are caregivers' rights to make medical decisions on behalf of their children absolute? Decision-making in pediatrics needs a shared approach where children, families, and healthcare multidisciplinary teams cooperate to reach a consensus on decisions in patients' best interests. This “shared” approach is fundamental in pediatric oncology to minimize children's medical and psychosocial impact (17).

The assessment of decision-making capacity for treatment and competence to consent in minors is still far to be validated (18). The acquisition of informed consent is delegated to parents or caregivers, often regardless of their ability to consent to treatment. Since children's capacities develop with growth and experience, their involvement in decisions regarding their health must also increase (19). The involvement of the minor in the diagnostic and therapeutic decision-making process is not solely based on patients' age or ability to read and write and should reduce the stress related to procedures and treatments (20). Some studies have attempted to apply to minors the MacCAT-CR and the MacCAT-T scale in various settings, including the neuropsychiatric one, with surprising results, as adolescents are likely to show the same results as adults (21). Nevertheless, these tools do not have external validation and further research is needed. Finally, all studies regarding this topic emphasize the need to implement communication between minors and parents/caregivers for therapeutic decisions (10, 22).

The outbreak of the COVID-19 pandemic addressed a challenging debate about the administration of new anti-covid vaccines for minors (23). There is a substantial heterogeneity standpoint on this topic in Western Countries. Vaccinating minors to protect them from the COVID-19 virus requires parental permission; the question arises concerning minors' decision-making capacity to consent to such preventive treatment by tracking adults' decisions. In this respect, Morgan et al. suggested a “guide” based on age groups to the consent of minors for the vaccine: is this age-based assessment sufficient? Is it enough to explain to children the benefits and burdens of procedure about a vaccine tested first? One more reason to promote research on assessing children's decision-making capacity for treatment (24).

### Healthcare systems and multi-level assistance

In addition to these intrinsic factors, the setting of children's care regarding safety is also essential. Currently, the child healthcare system has different intervention levels. Family pediatricians deliver primary care; secondary care is provided in pediatric departments in general hospitals. Children's hospitals were designed to provide optimal care and a multidisciplinary approach to patients and their families (25). Settings in which patients had the most complex needs imply a higher error rate compared with ambulatorial context. The Emergency Department is the place in the pediatric Children's Hospital, where multiple factors related to staff, patients, family members, and pre-hospital communication contribute to various diagnostic and medication mistakes (26).

The healthcare activities carried out in these units often involve multiple urgent interventions for high-risk patients in a complex and stressful environment that exposes children to high frequencies of errors. Applying clinical risk management tools and methodologies changed pediatric intensive care units, reducing medical errors and adverse events and organizational, clinical, and economic impacts, contributing to safety and overall healthcare quality (27). The integrated use of proactive and reactive methods had a growing trend over time, similarly to the increasing use of proactive tools highlighted in two systematic reviews (28, 29).

The pandemic outbreak disclosed the shortages of primary care organizations resulting in inappropriate hospitalizations for non-urgent diseases, thus increasing the risk of errors and unintended harm. Many studies reported a drastic reduction in access to emergency pediatric wards, thus emerging the often inappropriate use of these services (30). The overcrowding of the emergency-urgency department, already characterized by a greater incidence of medical errors, has an even more negative impact on care safety. Therefore, it is evident the need to build a functioning network for the care of the pediatric child, especially concerning primary care (31).

But what about other settings? Although it is evident that some departments are more exposed to risk than others, no hospital ward is risk-free. An interesting article by Lynne Warda reviewed the risks of injury to children in the hospital setting, identifying that those life-threatening hazards mainly concern beds or cribs, concluding that although safety considerations are well entrenched in occupational health and safety, the needs of children are typically not addressed (32). Children require healthcare focusing on their needs. They require more time and specificities for their care; for this reason, these must be treated in children's hospitals where every detail is thought out to optimize patient safety and well-being, from the facilities to the staff specially trained for pediatric care (12).

### The lack of guidelines and pediatric trials

Another pediatric “silent” crisis, as defined by Corinna Rea, concerns the lack of children's guidelines (13). Some countries favor a more centralized approach to guideline production. Others have a decentralized one, such as the US, where the American Academy of Pediatrics plays the most crucial role in developing guidelines. Decentralization seems to give rise to recommendations' production that may be overlapped, with the consequent detriment of their quality. Moreover, while there is a continuous demand for new guidelines, little importance is given to updating the old ones. Many of these results expired with all their negative consequences: the implementation of obsolete practices that can affect the quality and safety of care and distrust of clinicians and minors' families about the value of outdated guidelines. It is desirable to implement reviewing and validating procedures with greater urgency for the pediatric ones, whose development should be centralized and entrusted to a single organization. Moreover, in a broader perspective, to solve the lack of guidelines and good clinical practices, health practitioners should acquire the so-called “living guidelines” approach for ensuring inpatient high-level quality care: new evidence and recommendations are constantly monitored and possibly updated. A dynamic review approach should ensure the application of the best available practices to the pediatric population, assuring them of high-level quality care to prevent clinical risk (12).

Many barriers exist to conducting pediatric trials, so many therapies used in pediatrics are not evidence-based (15). On the other hand, recent legislation has aimed to stimulate pediatric research and drug labeling (33). In such context, the off-label use of COVID-19 vaccines rises peculiar ethical and legal concerns, as many scientific organizations cautioned against it (34).

### The importance of a safety culture

In the complex working environment of Children's Hospitals, it is necessary to improve the so-called “safety culture.” It consists, for professionals, in understanding e recognizing problems and risks in safety, understanding that the practice has value both for them and patients. It is also imperative for the hospital to have its safety policies, encouraging tailored and procedures baseline numbers about the safety problems before implementation (35, 36).

In light of these areas of vulnerability, pediatric clinical governance programs deserve specific attention. Several measures and tools could be implemented to reduce errors, promoting the so-called “safety culture”. The efforts to be done can be synthesized in these points:

- Special attention should be paid to training new healthcare professionals and integrating patient safety into ongoing medical education.
- Studies issuing pediatric patient safety should be promoted to improve working knowledge of children's patient safety issues throughout the pediatric community, especially in searching for a validated scale assessing the decision-making capacity of minors.
- Hospitals' safety policies should implement appropriate local procedures and train multidisciplinary teams to seek quality improvement, with a greater focus on the pediatric setting.
- The development process of pediatric guidelines and recommendations should be promoted.

It is our view that one of the best tools for implementing a “safety culture” is the so-called “clinical pathway”, which aims to standardize care processes and improve outcomes without increasing costs or compromising quality. Specifically, clinical pathways aim to integrate evidence into clinical practice and optimize patient outcomes while improving efficiency by translating national guidelines and the latest evidence into the local context. Multidisciplinarity and collaboration between all healthcare professionals are fundamental in the drafting and reviewing process of clinical pathways, also because they measurably improve many aspects of care for pediatric patients. As for guidelines, the spread of clinical pathways should be promoted, and where possible, it would be helpful to create a database to allow their dissemination in as many hospitals as possible and promote collaboration among these.

There is still a long way to go, but multidisciplinarity, the education of young professionals in the culture of safety, and the support of research in less explored fields are the keys to developing a safer healthcare system for pediatric patients.

## Author contributions

SN and BS conceived the manuscript. SN, DF, MB, and GM wrote the manuscript. All authors contributed to the article and approved the submitted version.

## Conflict of interest

The authors declare that the research was conducted in the absence of any commercial or financial relationships that could be construed as a potential conflict of interest.

## Publisher's note

All claims expressed in this article are solely those of the authors and do not necessarily represent those of their affiliated organizations, or those of the publisher, the editors and the reviewers. Any product that may be evaluated in this article, or claim that may be made by its manufacturer, is not guaranteed or endorsed by the publisher.
